# Focusing the spotlight on GSK-3 in aging

**DOI:** 10.18632/aging.100568

**Published:** 2013-06-20

**Authors:** Jibin Zhou, Thomas Force

**Affiliations:** Center for Translational Medicine Temple University School of Medicine Philadelphia, Pennsylvania, USA

Aging/senescence occurs at both the level of the whole organism and the individual cell. Organismal aging is usually defined as the progressive loss of function accompanied by decreasing fertility and increasing mortality with advancing age [1]. Cellular senescence refers to the permanent arrest of cell division, which is characterized by several cellular markers, identifying senescent cells *in vitro* and *in vivo* [2]. These include altered cellular morphology, increased activity of SA-β-GAL, accumulation of DNA damage foci, accumulation of senescence-associated heterochromatic foci (SAHF) [3] and promyelocytic leukemia protein nuclear bodies (PML-NBs) [4], chromosomal instability, and induction of an inflammatory secretome. Aging affects all organisms but to date, it is still unclear whether aging is triggered by genetic programs and/or evolutionary processes (Figure 1).

**Figure 1:**
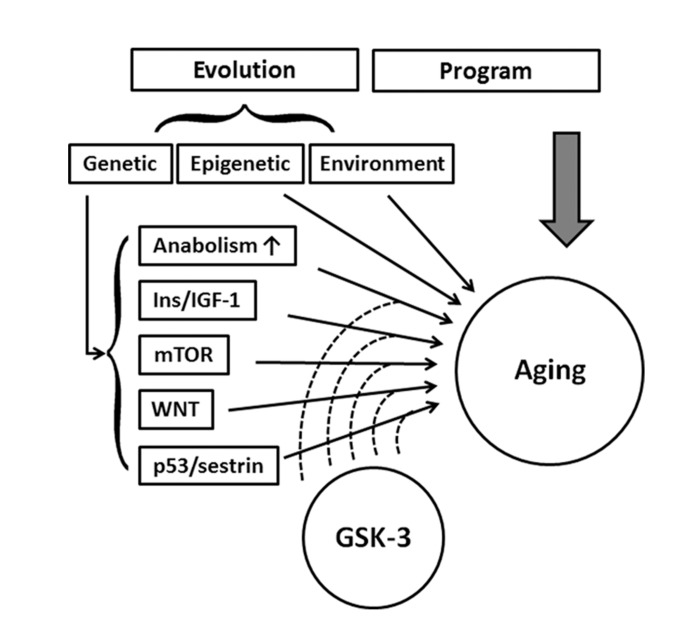
Schematic drawing represents the signaling pathways and proteins that have been reported to influence aging are regulated by GSK-3s, implying GSK-3 is a crucial regulator in the aging process.

A unifying theory that explains aging has not as yet emerged. That said, most scientists believe that just as with other biological processes, aging is regulated by conserved signaling pathways. Recently, the JCI commissioned a review series that focused on fundamental cellular mechanisms of aging and their relationship to human disease [5]. These mechanisms, that are not mutually exclusive, include shortened telomeres, the induction of the senescence-associated secretory phenotype (SASP), regulation of metabolism by sirtuins, altered mitochondrial metabolism, the mechanistic target of rapamycin (mTOR) signaling pathway and longevity, aging and immune system dysfunction, and aging-associated changes in pancreatic β-cells [5]. Thus, aging is, indeed, a complex biological process controlled by multiple genetic, epigenetic and environmental factors.

The glycogen synthase kinase-3 (GSK-3) family of serine/threonine kinases was first purified, and then later cloned, by Woodgett, Cohen and colleagues in the 1980s. It was found to phosphorylate, and negatively regulate, glycogen synthase, the rate-limiting enzyme in glycogen synthesis [6, 7]. However, very few enzymes exert as broad a regulatory influence on cellular functions as do the two isoforms of GSK-3(α and β). The substrates that are phosphorylated by GSK-3s can be classified into four categories: metabolic enzymes, signaling molecules, structural proteins, and transcription factors, typically involved in regulating cell proliferation & differentiation; cellular metabolism, cell survival and cell cycle regulation. Additionally, GSK-3 has been linked to several chronic diseases, including diabetes and Alzheimer disease. Nevertheless, it was not clear whether GSK-3 might regulate aging.

Our recent work seems to clearly implicate GSK-3, and specifically the α isoform, in aging [8]. Through targeting GSK-3α in the mouse, we found accelerated development of age-related pathologies in multiple organ systems. These included accelerated aging in the bone/skeletal system, leading to severe degenerative joint disease that was accompanied by increased inflammatory cytokines. The gut and liver also showed clear signs of accelerated aging. But the most striking findings were seen in the heart and skeletal muscle (i.e. striated muscle). These organ systems developed profound hypertrophy and dysfunction. Notably, on H and E staining and transmission EM, we saw innumerable structurally abnormal organelles, in particular (but not limited to) disrupted mitochondria. The profound nature of this suggested that the KOs were unable to clear these damaged organelles, possibly implicating dysfunctional autophagy. We confirmed that KO of GSK-3α markedly activated mTOR, and knowing that mTOR suppresses autophagy, we asked if autophagy was dysregulated. We confirmed that it was. The key remaining question was whether this dysregulation of autophagy was leading to (or at least contributing to) the abnormalities of striated muscle. We employed a second generation inhibitor of mTORC1, everolimus, and found that both cardiac contractile abnormalities and skeletal muscle abnormalities were largely corrected. It remains to be seen whether the numerous other organ systems that we found to be dysfunctional in the absence of GSK-3α will also be corrected by mTORC1 inhibition.

Given the number of signaling pathways that both influence aging and are regulated, at least in part, by GSK-3, combined with our more preliminary findings with degenerative joint disease, and abnormalities of the gut, and liver, we believe that additional rolls will be identified for GSK-3 in aging. Understanding the influence of GSK-3s in aging is likely to provide important insights that will not only guide investigation of the molecular and cellular basis of aging, but may also help to identify novel treatment strategies targeting these pathways.

## References

[R1] Kirkwood TB, Austad SN (2000). Nature.

[R2] Sikora E (2011). Ageing Res Rev.

[R3] Zhang R (2005). Dev Cell.

[R4] Bernardi R, Pandolfi PP (2007). Nat Rev Mol Cell Biol.

[R5] Newgard CB, Sharpless NE (2013). J Clin Invest.

[R6] Embi N (1980). Eur J Biochem.

[R7] Woodgett JR (1982). FEBS Lett.

[R8] Zhou J (2013). J Clin Invest.

